# Bone marrow derived mesenchymal stem cells pretreated with erythropoietin accelerate the repair of acute kidney injury

**DOI:** 10.1186/s13578-020-00492-2

**Published:** 2020-11-16

**Authors:** Song Zhou, Yu-ming Qiao, Yong-guang Liu, Ding Liu, Jian-min Hu, Jun Liao, Min Li, Ying Guo, Li-pei Fan, Liu-Yang Li, Ming Zhao

**Affiliations:** Department of Organ Transplantation, Zhujiang Hospital, Southern Medical University, No. 253, Industrial Avenue, Haizhu District, Guangzhou, 510280 Guangdong Province China

**Keywords:** Acute kidney injury, Bone marrow derived mesenchymal stem cells, Erythropoietin, Lung entrapment, SIRT1

## Abstract

**Background:**

Mesenchymal stem cells (MSCs) represent a promising treatment option for acute kidney injury (AKI). The main drawbacks of MSCs therapy, including the lack of specific homing after systemic infusion and early cell death in the inflammatory microenvironment, directly affect the therapeutic efficacy of MSCs. Erythropoietin (EPO)-preconditioning of MSCs promotes their therapeutic effect, however, the underlying mechanism remains unknown. In this study, we sought to investigate the efficacy and mechanism of EPO in bone marrow derived mesenchymal stem cells (BMSCs) for AKI treatment.

**Results:**

We found that incubation of BMSCs with ischemia/reperfusion(I/R)-induced AKI kidney homogenate supernatant (KHS) caused apoptosis in BMSCs, which was decreased by EPO pretreatment, indicating that EPO protected the cells from apoptosis. Further, we showed that EPO up-regulated silent information regulator 1 (SIRT1) and Bcl-2 expression and down-regulated p53 expression. This effect was partially reversed by SIRT1 siRNA intervention. The anti-apoptotic effect of EPO in pretreated BMSCs may be mediated through the SIRT1 pathway. In a rat AKI model, 24 h after intravenous infusion, GFP-BMSCs were predominantly located in the lungs. However, EPO pretreatment reduced the lung entrapment of BMSCs and increased their distribution in the target organs. AKI rats infused with EPO-BMSCs had significantly lower levels of serum IL-1β and TNF-α, and a significantly higher level of IL-10 as compared to rats infused with untreated BMSCs. The administration of EPO-BMSCs after reperfusion reduced serum creatinine, blood urea nitrogen, and pathological scores in I/R-AKI rats more effectively than BMSCs treatment did.

**Conclusions:**

Our data suggest that EPO pretreatment enhances the efficacy of BMSCs to improve the renal function and pathological presentation of I/R-AKI rats.

## Background

Acute kidney injury (AKI) is one of the most clinically impactful diseases with high morbidity and mortality [1, 2]. Multiple injuries such as those resulting from sepsis, ischemia/reperfusion (I/R), and drug administration may induce AKI. I/R injury is a major cause of human AKI, which is associated with tubular necrosis, cast formation, tubular dilation, loss of brush border, and inflammation [3]. AKI remains a worldwide public health concern due to the increased risk of subsequent development of chronic kidney disease (CKD) [4]. The annual medical expenses associated with AKI treatment places a heavy burden on the public health care system, and yet AKI lacks an established treatment strategy. Therefore, there is an urgent need to find innovative and effective therapeutic strategies for treating AKI. The application of mesenchymal stem cells (MSCs) has been suggested as a promising treatment strategy for AKI [5, 6].

In recent years, MSCs have become an area of intense research in the field of stem cell therapy. MSCs are adherent, fibroblast-like cells derived from different tissues and organs, including bone marrow, umbilical cord blood, adipose tissue, and solid organs that have the potential for multidirectional differentiation and self-renewal. The tissue reparative/regenerative and immunoregulatory properties of MSCs render them as promising candidates for cell therapy and tissue regeneration. A large number of basic science studies and clinical trials have demonstrated the safety, feasibility, and effectiveness of MSCs in the treatment of myocardial infarction, spinal cord injury, diabetes, and kidney disease [7–9]. MSCs exhibit renoprotective effects in both acute and chronic kidney injury. These effects seem to be associated with immunomodulation, anti-apoptotic effects, and reduction of disease-related inflammation [10, 11].

The main drawbacks of MSCs therapy are early pulmonary entrapment and the lack of specific homing to target tissues following systemic infusion [12, 13]. Even when the MSCs reach the target organ, many of the cells undergo apoptosis mainly due to the inhospitable local microenvironmental conditions, such as hypoxia, oxidative stress, and inflammation, which directly affect the therapeutic efficacy of MSCs [14, 15]. Application of pretreated MSCs is a novel strategy to enhance the capacity of MSCs to migrate and promote tissue repair in AKI [16, 17].

EPO is a glycoprotein hormone that promotes the proliferation and differentiation of bone marrow hematopoietic stem cells (HSCs) and the hematopoietic function of bone marrow through EPO receptor (EpoR) signaling. BMSCs and bone marrow HSCs are homologous and express EpoR. Studies have shown that EPO is suitable for the treatment of a variety of diseases, including cerebral ischemia, myocardial infarction, chronic congestive heart failure, and renal injury [18, 19]. In our previous study, we demonstrated that pretreatment with 500 IU/ml erythropoietin (EPO) for 48 h prior to infusion markedly increased the homing and healing abilities of bone marrow-derived mesenchymal stem cells (BMSCs). These BMSCs significantly inhibited the apoptosis induced by cyclosporine A (CsA) toxicity in HK2 cells. Moreover, the single infusion treatment with EPO-BMSCs significantly improved the renal function in CsA-induced chronic toxic renal injury, and promoted the repair of renal fibrosis in rats [20].

In this study, we sought to investigate the efficacy and mechanism of EPO pretreatment on BMSCs for the treatment of AKI. We demonstrate that EPO promotes the survival of transplanted BMSCs in an inflammatory microenvironment by stimulating the SIRT1-dependent pathway.

## Results

### The characterization of EPO-treated BMSCs

**Fig. 1:**
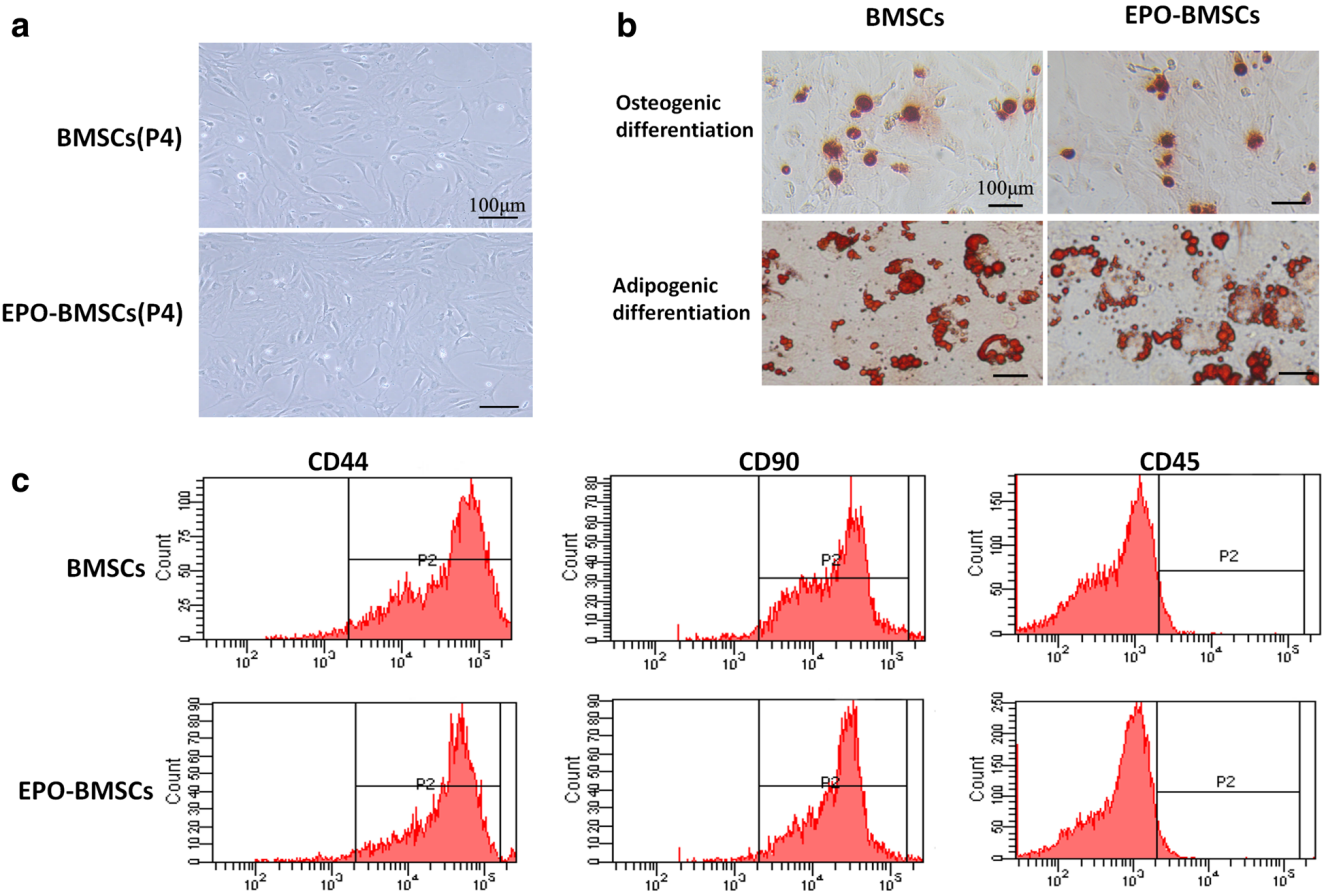
Cell culture and characterization of BMSCs. **a** Images of P4 BMSCs and EPO-treated BMSCs as observed under phase contrast microscope. **b** BMSCs and EPO-BMSCs differentiated into osteoblasts and adipocytes. **c** Flow cytometric analysis confirmed that untreated and EPO-treated cells were CD45-negative and positive for the phenotypic markers CD90 and CD44

The passage 4 (P4) BMSCs and EPO-treated BMSCs were observed under the phase contrast microscope (Fig. 1a). Both untreated and EPO-treated BMSCs were capable of osteogenic and adipogenic differentiation when cultured in the appropriate inducing media. After 3 weeks of incubation, BMSCs and EPO-BMSCs differentiated into osteoblasts and adipocytes (Fig. 1b). Flow cytometric analysis confirmed that both groups of cells were CD45-negative but positive for the phenotypic markers CD90 and CD44 (Fig. 1c). Both groups of cells maintained their stem cell characteristics.

### EPO-pretreated inhibited AKI-KHS-induced apoptosis in BMSCs

**Fig. 2:**
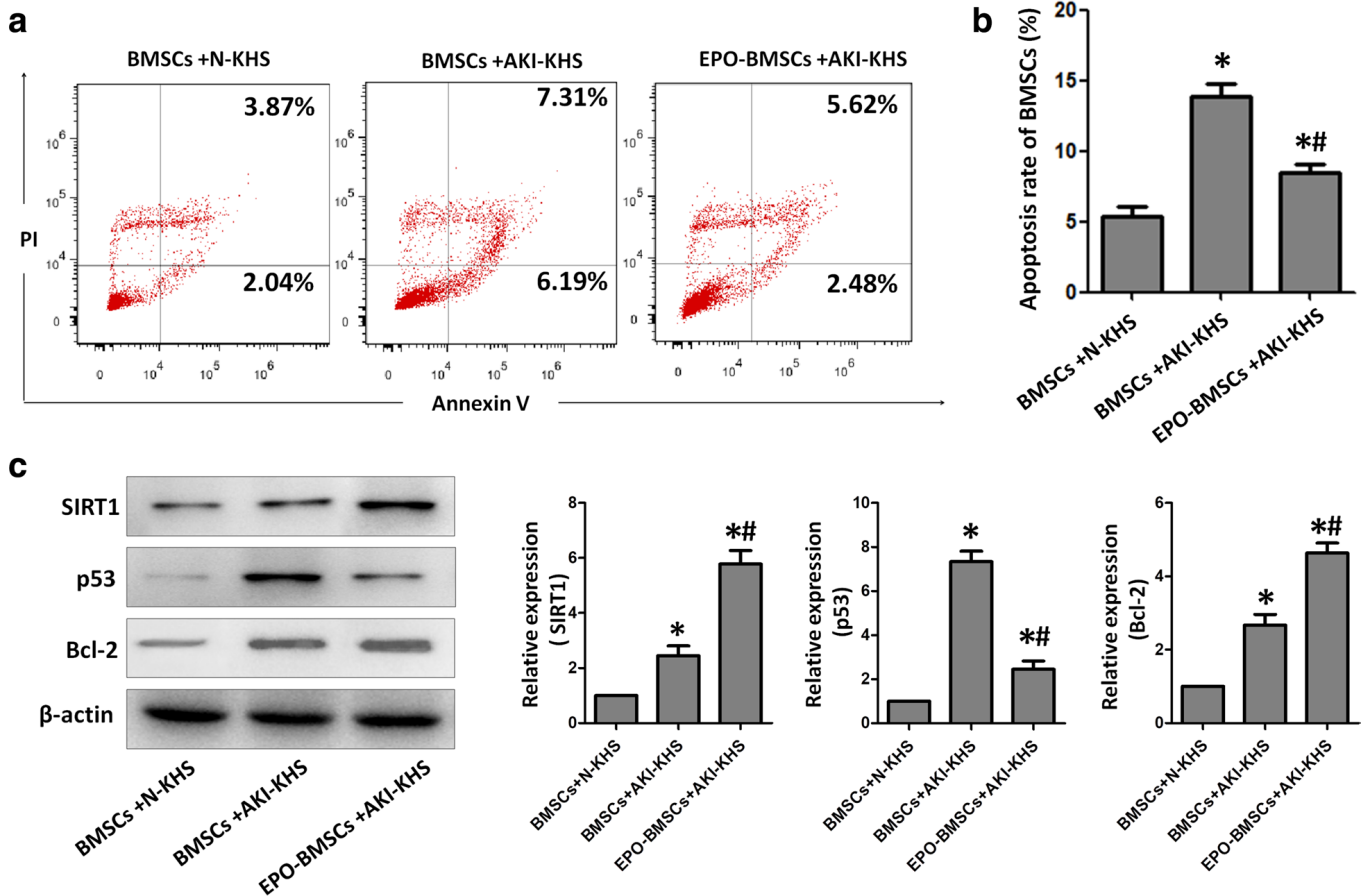
AKI-KHS induced apoptosis in BMSCs. **a** Flow cytometric analysis was performed 24 h after culturing to assess apoptosis in each group. **b** Apoptotic rates in each group were analyzed. **c** Anti-apoptotic factor Bcl-2, apoptotic factor p53, and SIRT1 were examined using western blot analyses. *p < 0.05 versus BMSCs + N-KHS,#p < 0.05 versus BMSCs + AKI-KHS

To study the effect of EPO on BMSCs, we established AKI-KHS-induced in vitro injury model. After co-culture with AKI-KHS for 24 h, the EPO-pretreated attenuated the apoptosis rate of BMSCs significantly. BMSCs, EPO-BMSCs were incubated with AKI-KHS (or N-KHS), after culture for 24 h, flow cytometric analysis was performed to assess apoptosis in each group (Fig. 2a). The apoptotic rates were significantly higher in the BMSCs + AKI-KHS and EPO-BMSCs + AKI-KHS groups when compared with that of the control group (p < 0.05). AKI-KHS induced apoptosis in BMSCs, but EPO pretreatment protected the cells from this apoptotic effect (p < 0.05) (Fig. 2b).

Protein was extracted from cultured cells and examined using western blot analyses (Fig. 2c). SIRT1 and Bcl-2 protein expression in both BMSCs + AKI-KHS and EPO-BMSCs + AKI-KHS groups were significantly increased compared to that in the control group (p < 0.05), especially in the EPO-BMSCs + AKI-KHS group (p < 0.05). The expression of p53 protein was significantly higher in the BMSCs + AKI-KHS group compared to the other two groups (p < 0.05).

### EPO exerted protective effect on BMSCs subjected AKI-KHS via activating SIRT1-p53 signaling

**Fig. 3:**
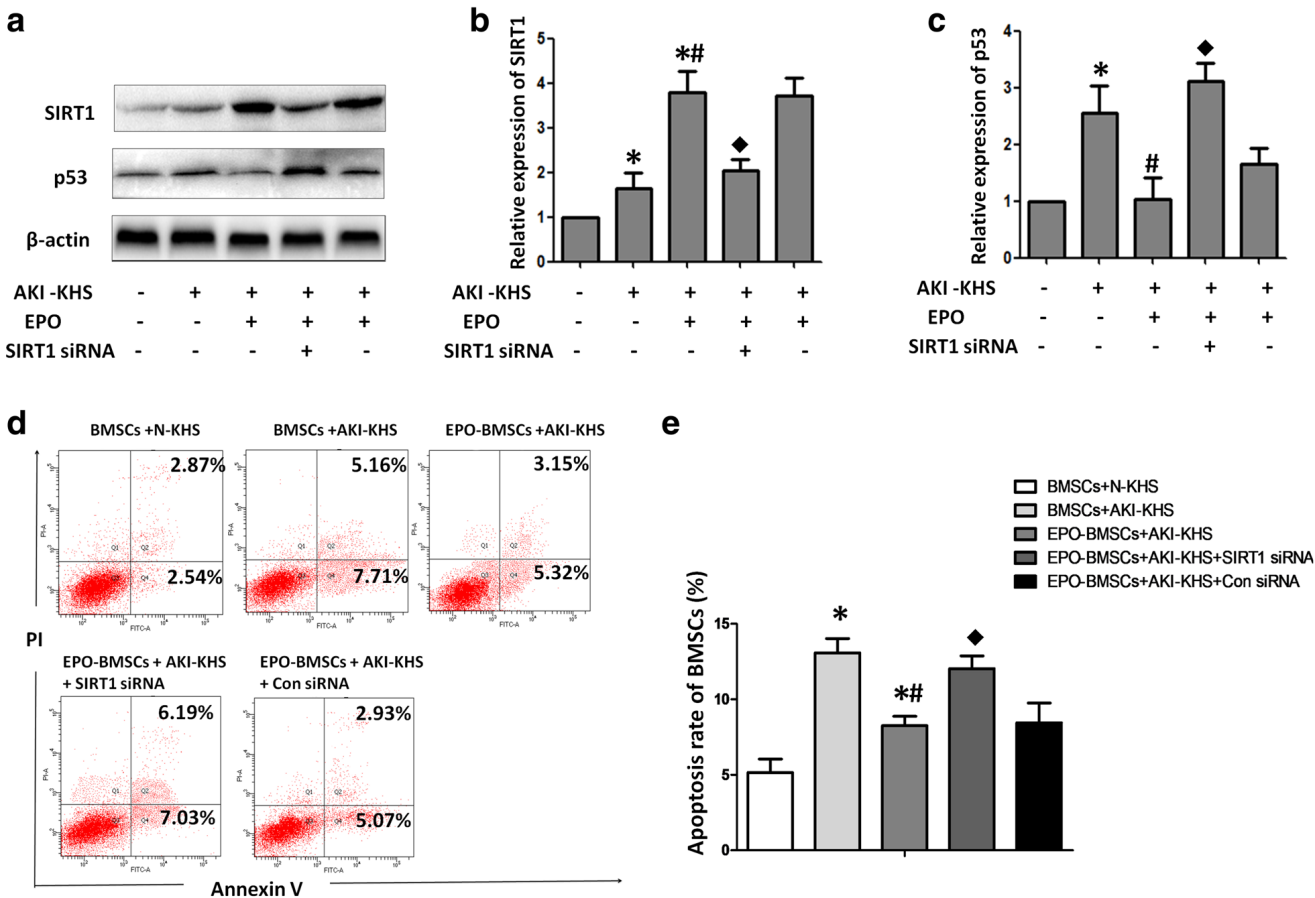
EPO exerted protective effect on BMSCs subjected AKI-KHS via activating SIRT1-p53 signaling. **a** Western blot analysis of the effect of EPO on the expression of SIRT1 and p53. **b** Relative expression of SIRT1. **c** Relative expression of p53. **d** Flow cytometric analysis was performed to assess apoptosis in each group. **e** Apoptotic rates in each group were analyzed. *p < 0.05 versus BMSCs + N-KHS, ^#^p < 0.05 versus BMSCs + AKI-KHS, ^◆^p < 0.05 versus EPO-BMSCs + AKI-KHS

Results of the flow cytometric analysis demonstrated that AKI-KHS-treated had an adverse effect on the survival of BMSCs while the EPO pretreatment improved BMSCs survival in the AKI microenvironment. To further investigate the anti-apoptotic mechanism of EPO pretreatment of the BMSCs, we analyzed the expression of the apoptotic factor p53, and SIRT1. The expression of SIRT1 in the EPO-BMSCs + AKI-KHS group was significantly increased compared with that in the BMSCs + AKI-KHS group (p < 0.05), and the expression of p53 was significantly higher in the BMSCs + AKI-KHS group than in the EPO-BMSCs + AKI-KHS group (p < 0.05). The effect of EPO on the expression of SIRT1 and p53 was partially reversed by SIRT1 siRNA intervention (Fig. 3a–c).

To prove the anti-apoptotic effect of EPO on BMSCs though SIRT1, we established AKI-KHS-induced in vitro injury model again. BMSCs, EPO-BMSCs, EPO-BMSCs + SIRT1 siRNA, and EPO-BMSCs + Con siRNA were incubated with AKI-KHS, and BMSCs incubated with N-KHS was used as control. After culture for 24 h, flow cytometric analysis was performed to assess apoptosis in each group (Fig. 3d). The anti-apoptotic effect of EPO was partially reversed by SIRT1 siRNA intervention (Fig. 3e).

### Pretreatment with EPO markedly increased the homing abilities of BMSCs

**Fig. 4:**
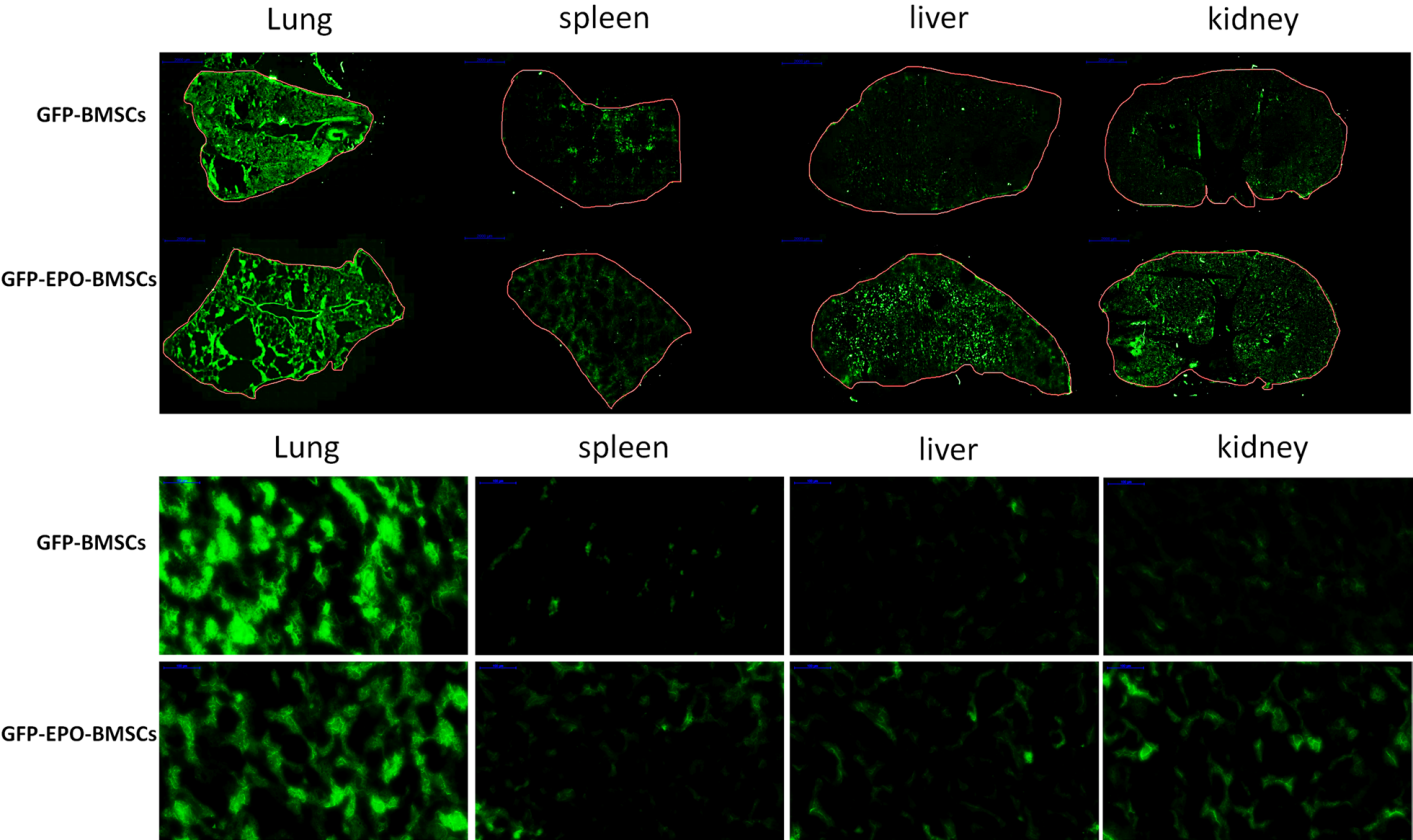
GFP fluorescence images of frozen sections from the lung, spleen, liver and kidney of rats. Sections were prepared 24 h after intravenous infusion of BMSCs and EPO-BMSCs

In our previous study, we demonstrated that pretreatment with 500 IU/ml EPO for 48 h prior to infusion markedly increased the homing abilities of BMSCs. To verify the in vivo distribution of BMSCs after intravenous infusion, we prepared fast-frozen sections from the kidney, lung, spleen, and liver 24 h after GFP-BMSCs and GFP-EPO-BMSCs infusion. Our data showed that 24 h after intravenous infusion, a majority of the GFP-BMSCs were trapped in the lungs, with minimal fluorescence detected in the spleen, liver, and kidneys. In the GFP-EPO-BMSCs group, the fluorescence intensity was significantly lower in the lungs but higher in other organs, especially the kidneys, compared with the corresponding fluorescence intensities in the GFP-BMSCs group. GFP-BMSCs and GFP-EPO-BMSCs were predominantly detected in the lungs, but EPO preconditioning reduced the lung entrapment of BMSCs and increased their distribution in the target organs (Fig. 4).

### EPO-BMSCs improved renal function after I/R injury

**Fig. 5:**
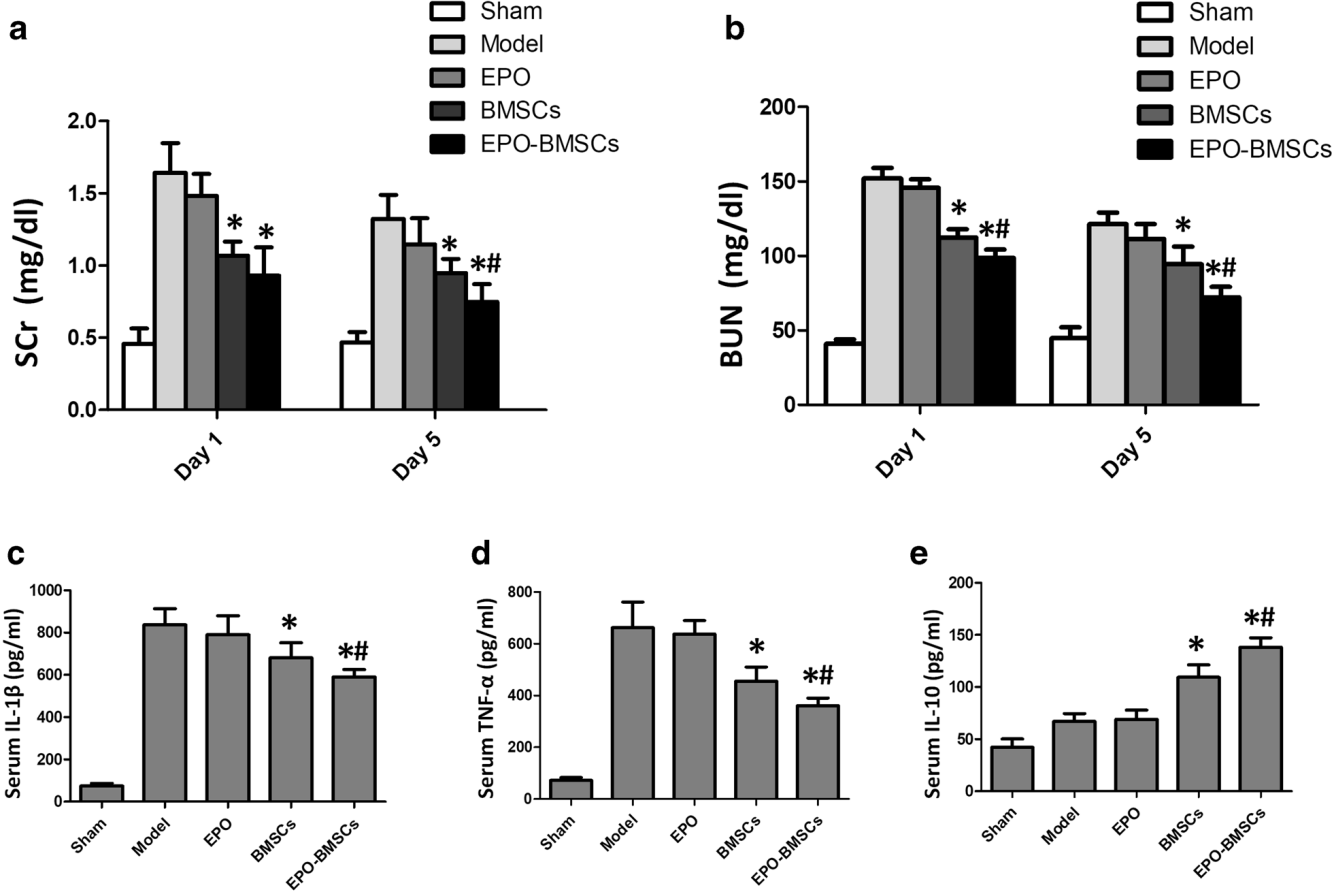
The effects of BMSCs and EPO-BMSCs treatment on AKI rats. Serum level of **a** SCr and **b** BUN measured on day 1 and 5 after treatment. Serum level of the pro-inflammatory cytokines **c** IL-1β and **d** TNF-α and the anti-inflammatory cytokine **e** IL-10 measured by ELISA 24 h after the indicated treatment.*p < 0.05 versus Model group, ^#^p < 0.05 versus BMSCs group

To compare the effects of BMSCs and EPO-BMSCs on AKI rats, we measured the levels of blood urea nitrogen (BUN) and serum creatinine (SCr) on day 1 and 5 after treatment. Treatment of AKI rats with EPO, BMSCs, and EPO-BMSCs showed varying therapeutic effects. All three treatment groups reduced the level of BUN and SCr on day 1 and 5 compared with the model group. However, only BMSCs and EPO-BMSCs treatment showed a significant effect (p < 0.05). Rats in the EPO-BMSCs group had significantly lower BUN and SCr levels than those in the BMSCs group on day 5 (p < 0.05, Fig. 5a, b).

### EPO-BMSCs suppressed inflammatory response after I/R injury

Twenty-four hours after the treatment of AKI rats, serum concentrations of the inflammatory factors were measured by ELISA. There was a significant increase in the level of IL-1β, TNF-α, and IL-10 1 day after the induction of AKI. AKI rats that underwent EPO-BMSCs infusion had significantly lower serum IL-1β and TNF-α levels and a significantly higher IL-10 serum level than those in AKI rats that received BMSCs infusion. The serum levels of the cytokines in the EPO group did not significantly differ from those in the model group (Fig. 5c–e).

### EPO-BMSCs enhanced the effect to ameliorate pathological injuries

**Fig. 6:**
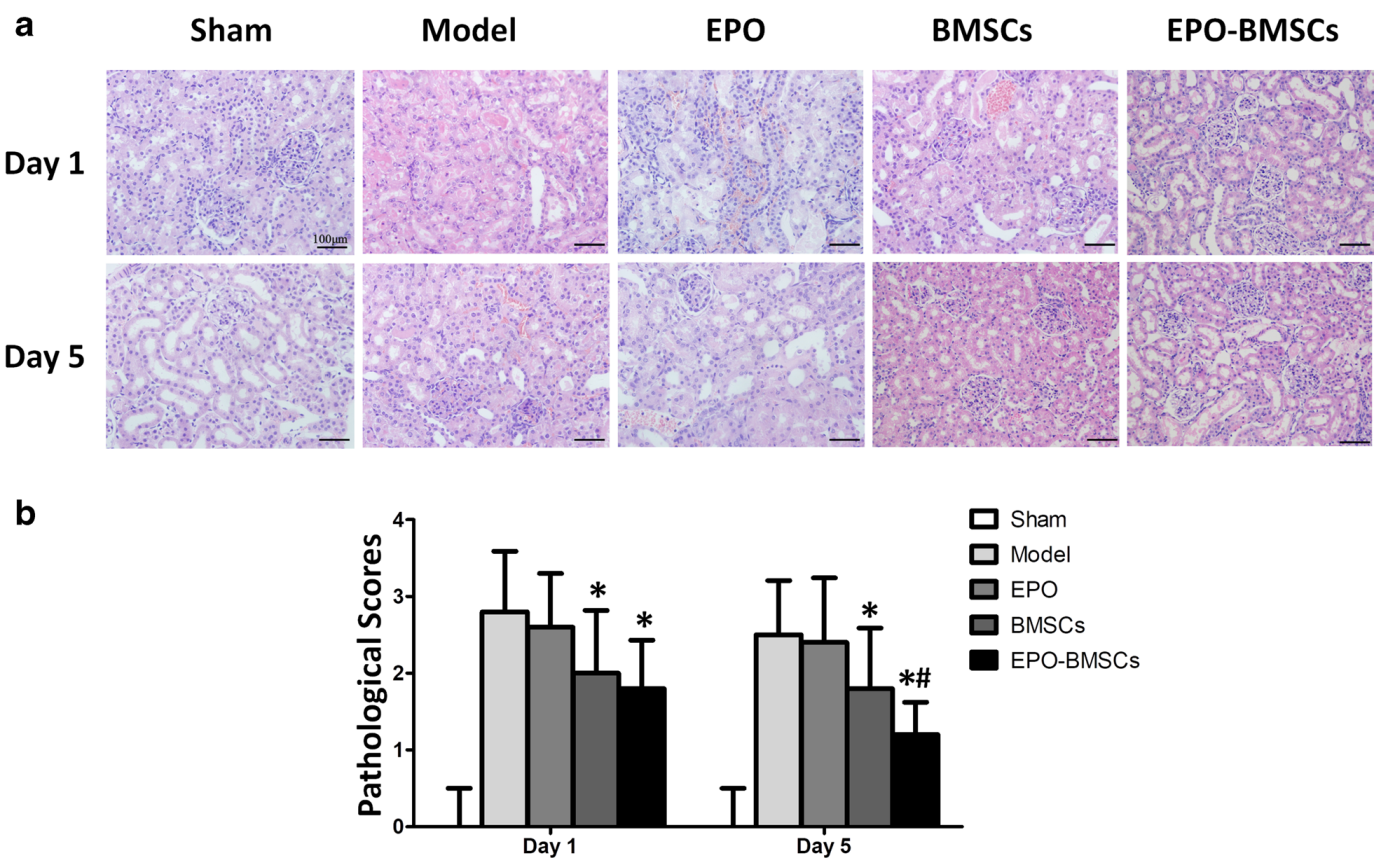
Histological analysis. **a** Kidney sections stained with HE on day 1 and 5 after each treatment. **b** Pathological score of each group one and five days after each treatment.*p < 0.05 versus model group; ^#^p < 0.05 versus BMSCs group

Hematoxylin and eosin (HE) staining of kidney sections revealed tubular necrosis, cast formation, tubular dilation, and loss of brush border in the AKI model group (Fig. 6a). These pathological injuries were assessed using the pathological scores obtained after treatment with EPO, BMSCs, and EPO-BMSCs on day 1 and 5. The pathological scores in the BMSCs and EPO-BMSCs treatment groups were significantly lower than those in the model group 1 and 5 days after treatment (p < 0.05). Both BMSCs and EPO-BMSCs infusion reduced tubular injury; however, EPO-BMSCs administration improved tubular injury significantly more on day 5 as compared to infusion with BMSCs (Fig. 6b).

## Discussion

In the present study, we reveal novel insights regarding the efficacy and mechanism of EPO-BMSCs in I/R-AKI. Our findings showed that I/R-AKI microenvironment caused apoptosis in BMSCs, but EPO pretreatment protected the cells from this apoptotic effect. We found that EPO up-regulated SIRT1 and Bcl-2 expression and down-regulated p53 expression in the AKI model. The anti-apoptotic effect of EPO may be mediated through the SIRT1 pathway. EPO pretreatment reduced pulmonary entrapment of BMSCs and increased the number of cells reaching the target organs. Furthermore, EPO pretreatment reduced the expression of disease-related inflammatory cytokines. AKI rats that underwent EPO-BMSCs infusion had significantly lower serum IL-1β and TNF-α levels, and a significantly higher IL-10 level than those in rats treated with BMSCs. Our data suggest that EPO pretreatment enhances the efficacy of BMSCs to improve renal function and pathological presentation of I/R-AKI rats.

The protective effect of MSCs in acute and chronic renal injury may be through a paracrine/autocrine mechanism, which is related to immune regulation, anti-apoptosis, and reduction of disease-related inflammation. Several studies have shown that the survival and retention of MSCs in target organs or tissues is closely related to the therapeutic effect mediated by MSCs. The main drawbacks of MSCs therapy are the early pulmonary entrapment and the lack of specific homing after systemic infusion. Most of the MSCs undergo cell death after transplantation, mainly due to an adverse microenvironment, directly affecting the therapeutic efficacy of MSCs [14, 15].

Increasing the dose of MSCs infused represents a viable option to achieve better therapeutic effects; however, this is accompanied by some side effects, such as microvascular embolization and the potential risk of long-term tumor growth. Therefore, current studies on MSCs-related therapies have focused on lower infusion doses to achieve the greatest therapeutic effect possible. Cell-free treatments, including microvesicles, exosomes, specific cytokines, miRNA, and the pretreatment of MSCs, represent viable alternatives to address the issue of long-term negative effects [21–23]. Pretreating MSCs is a novel strategy to enhance the capacity of MSCs to migrate and promote tissue repair in injured kidneys [24]. Pretreatment, before infusion, with cytokines such as transforming growth factor β1 (TGF-β1), interleukin-17A (IL-17A), and melatonin can increase the number of MSCs homing to the injured kidney, promote the recovery of renal function, and ameliorate renal impairments [3, 17, 25]. Similar results were obtained upon pretreating MSCs with EPO.

EPO is a glycoprotein hormone that promotes the proliferation and differentiation of bone marrow HSCs and the hematopoietic function of bone marrow through EpoR signaling. BMSCs and bone marrow HSCs are homologous and express EpoR. However, many other cell types, including neurons, endothelial cells, cardiomyocytes, and renal tubular cells also express the EpoR and respond to EPO treatment [26]. Studies have shown that EPO is suitable for the treatment of a variety of diseases, including cerebral ischemia, myocardial infarction, chronic congestive heart failure, and renal injury [18, 19]. Moreover, some studies suggest that overexpression of EPO in MSCs by gene-transfection could further enhance the therapy effect of MSCs [27, 28]. Compared with other pretreatments or strategies that improve the therapeutic effect of MSCs, the in vitro pretreatment with EPO has significant advantages in terms of clinical feasibility. First, EPO is a commonly used therapeutic drug with few side effects and is widely used in the clinical treatment of anemia, especially in patients with CKD. Second, it exhibits anti-oxidative and anti-inflammatory effects.

Our data showed that 24 h following intravenous infusion, GFP-BMSCs were predominantly located in the lungs. EPO preconditioning reduced the entrapment of BMSCs in the lungs and increased the distribution of GFP-BMSCs in the target organs. In our previous study, we showed that the pretreatment of BMSCs with an optimal concentration of EPO for an appropriate time induced a marked change in the proliferation rate and cytoskeletal rearrangement of BMSCs. After incubation with EPO, most of the cells exhibited parallelly-oriented filaments organized along the cell axis. We observed that C-X-C chemokine receptor type 4 (CXCR4), a pivotal mediator of migration and engraftment in MSCs, was up-regulated following EPO treatment, enhancing the migration ability of BMSCs [20].

Homing of MSCs to injured tissues is very critical in cell therapy. Various methods to apply MSCs exist, such as peripheral intravenous infusion, arterial infusion to the target organ, and local injection. Local injection increases the risk of bleeding and tissue injury, while direct arterial administration can result in occlusion and embolization of the target organs. Therefore, MSCs are mostly administered through a standard intravenous route. Pulmonary entrapment is a major problem after intravenous infusion of MSCs. Harting et al. [29] demonstrated that less than 4% of the infused cells were likely to traverse the pulmonary microvasculature and reach the arterial circulation, a phenomenon termed “pulmonary first-pass effect”, which limits the efficacy of this therapeutic approach. Some studies show that small microspheres (4–5 μm) could pass through the pulmonary system, whereas the majority of the large microspheres (20-μm) and MSCs (15–19 μm) are trapped within the lungs. Lung entrapment may be due to the small capillary size, large capillary network of the lungs, and strong adhesion properties of MSCs [30–32]. A variety of molecules may be involved in lung entrapment of systemically infused cells, and the composition of cell surface molecules, such as α4, α5, and α6 integrins, may affect the migratory behavior of the therapeutic cells [13, 32]. We found that after intravenous infusion, most of the stem cells were trapped within the lungs, but EPO pretreatment reduced pulmonary entrapment and increased the number of cells reaching the target organ.

We also found that EPO up-regulated SIRT1 and Bcl-2 expression and down-regulated p53 expression. SIRT1 is an NAD^+^-dependent deacetylase belonging to class III histone deacetylases and is known as the longevity protein in mammals. SIRT1 regulates a variety of cellular signaling pathways by modifying the acetylation status of target proteins including p53, members of the forkhead family of transcription factors (FOXO), and nuclear factor NF-κB. Following activation, SIRT1 participates in a plethora of cellular processes such as cell senescence, apoptosis, DNA damage repair, cell cycle, oxidative stress response, energy metabolism regulation, tumor generation, and other physiological and pathological processes [33–35]. Tumor suppressor protein, p53, plays a vital role in apoptotic signaling pathways, including membrane and mitochondrial apoptotic pathways, and affects the transcription and expression of many apoptosis-related cytokines in the nucleus [36]. SIRT1 reduces the transcriptional activity of p53 and inhibits p53-dependent cell apoptosis, caused by DNA damage [37]. In tumor studies, SIRT1 inhibited apoptosis in tumor cells by regulating p53 and Bcl-2 [38]. EPO has been demonstrated to impede DOX-induced cardiotoxicity by activating SIRT1, leading to enhancement of mitochondrial function [18]. Hong et al. [33] revealed that EPO alleviates hepatic steatosis by activating autophagy through SIRT1-dependent de-acetylation of LC3. SIRT1 plays an important role in maintaining the self-renewal and differentiation of MSCs, especially under stress conditions [39, 40], and also exhibits positive effects on senescence and apoptosis of MSCs [41, 42].

In our study, most of the BMSCs undergo cell death after transplantation, mainly due to the adverse local microenvironment. Therefore, improving cell survival and retention of BMSCs is pertinent to promote their therapeutic efficacy in AKI therapy. The observed beneficial effect of EPO pretreatment was associated with SIRT1 signaling activation, leading to the de-acetylation and subsequent inactivation of p53 and up-regulation of Bcl-2 expression, which in turn reduces apoptosis in BMSCs. These findings, therefore, demonstrate that the anti-apoptotic effect following EPO pretreatment of BMSCs may be mediated though the SIRT1 pathway.

The mechanism by which EPO-pretreated BMSCs accelerate the repair of AKI includes three facets. First, after incubation with EPO, most of the BMSCs exhibited parallelly oriented filaments organized along the cell axis and showed increased CXCR4 expression. These changes reduced the lung entrapment of BMSCs and increased their homing to target organs. Second, EPO up-regulated SIRT1 and Bcl-2 expression, and down-regulated p53 expression in BMSCs. SIRT1 inhibited apoptosis in BMSCs by regulating p53 and Bcl-2 expression. Third, the direct anti-inflammatory effect of EPO-BMSCs is also likely to be involved in the process. However, the present study has some limitations and did not elucidate the mechanism underlying EPO-mediated activation of SIRT1 signaling in BMSCs, which requires future investigations.

## Conclusion

In conclusion, EPO-BMSCs were more effective in reducing the levels of SCr and BUN, and the pathological scores in I/R-AKI rats after reperfusion when compared to untreated BMSCs. These results suggest that EPO pretreatment may potentially be a novel alternative to untreated BMSCs for the management of AKI.

## Materials and methods

### Cell culture

Sprague–Dawley (SD) bone marrow derived mesenchymal stem cells (BMSCs) (Cyagen Biosciences Inc., Guangzhou, China) were propagated at 37 °C under 5% CO_2_ in Dulbecco’s Modified Eagle Medium (DMEM, Gibco, Carlsbad, CA) cultured with 10% fetal bovine serum (FBS, Gibco) and 1% antibiotics (50 U mL^−1^ penicillin and 50 ug mL^−1^ streptomycin, Gibco). The passage 4(P4) cells were used for the in vitro experiments. SD-derived BMSCs(P3) (Cyagen Biosciences Inc., USA) that stably express GFP were propagated and the P5-GFP-BMSCs were used for in vivo experiments.

### Characterization of BMSCs incubated with EPO

BMSCs were trypsinized after incubation with or without EPO (500 IU/ml) for 48 h and washed three times with phosphate-buffered saline (PBS). Cell surface markers were examined by immunostaining with the following antibodies: phycoerythrin (PE)-conjugated anti-CD45 (BD Biosciences, San Jose, CA), fluorescein isothiocyanate (FITC)-conjugated anti-CD44 (BD Biosciences), and FITC-conjugated anti-CD90 (BD Biosciences) antibody. The labeled cells were analyzed using a flow cytometer (BD LSRFortessa™, Piscataway, NJ). Osteogenic and adipogenic differentiation potential of the cells in the two groups was examined according to the manufacturer’s protocol (Cyagen, China).

### I/R-AKI kidney homogenate supernatant (KHS) preparation

An AKI model was established using SD rats by clamping both renal pedicles for 45 min, followed by clamp-release to allow reperfusion. Both kidneys were harvested 60 min after reperfusion, cut into small pieces, and homogenized in PBS using a glass homogenizer to obtain a 20 g/l homogenate. The homogenate was then centrifuged at 20,000 rpm for 15 min at 4 °C. The supernatant was filtered through a 30-μm mesh-sized disposable sterile filter to obtain AKI-KHS, which was stored at − 80 °C until further use. Normal KHS (N-KHS) obtained from healthy SD rats was used as a control.

### AKI-KHS-induced apoptosis in BMSCs

To study the anti-apoptotic effect of BMSCs pretreated with EPO, we established AKI-KHS-induced in vitro injury model: BMSCs or EPO-BMSCs were incubated with AKI-KHS (or N-KHS). Cells were seeded at 4 x10^5^ cells/well in six-well plates in DMEM/F12 with 2% FBS. Transwell chambers with 0.4 μm pore size polycarbonate filter (Corning Incorporated, NY, USA) were introduced in the wells for the interventions. Three groups were set up: the control group (BMSCs + N-KHS group, BMSCs were plated in the 6-well plates and 1.5 ml N-KHS was added to the upper chamber); the BMSCs + AKI-KHS group (BMSCs were plated in the 6-well plates and 1.5 ml AKI-KHS was added to the upper chamber); and the EPO-BMSCs + AKI-KHS group (EPO-BMSCs were plated in the 6-well plates and 1.5 ml AKI-KHS was added to the upper chamber). All groups were incubated at 37 °C for 24 h in a humidified atmosphere with 5% CO_2_. Then, apoptosis of the BMSCs and EPO-BMSCs was analyzed by flow cytometry, the Annexin V-FITC apoptosis detection kit (Invitrogen, Frederick, MD, USA) was used to detect apoptotic cells according to the manufacturer’s protocol. Then cells were harvested subsequently for western blot analysis.

### Western blotting

After each treatment, the cells were washed twice with PBS, harvested, and the proteins were extracted. The following antibodies were used to analyze protein expression: anti-BCL-2 (1:1000, Santa Cruz, CA), p53 (1:1000, Santa Cruz), SIRT1 (1:1000, Santa Cruz), and anti-β-actin (1:2000, Bioworld, Shanghai, China). Protein bands were quantified by densitometry using an Alpha Innotech imaging system. Protein levels were normalized to β-actin expression using Image J analysis software.

### SiRNA transfection

Cells were transiently transfected with SIRT1 siRNA or control siRNA using Lipofectamine RNAiMAX reagent (Life Technologies, Carlsbad, CA) according to the manufacturer’s protocol. Cells were analyzed 36 h after siRNA transfection. The SIRT1 siRNA and control siRNA were synthesized by RiboBio technologies (RiboBio, Guangzhou, China). The sequence of SIRT1 siRNA was as follows: 5′-CACCUGAGUUGGAUGAUAUTT-3′ (sense) and 5′-AUAUCAUCCAACUAGGUGTT-3′ (antisense).

### Anti-apoptotic effect of BMSCs pretreated with EPO

To study the anti-apoptotic effect of BMSCs pretreated with EPO, we incubated BMSCs and EPO-BMSCs with AKI-KHS (or N-KHS). Five groups were set up: the BMSCs + N-KHS group was used as a control(BMSCs were plated in 6-well plates and 1.5 ml N-KHS was added to the upper chamber); the BMSCs + AKI-KHS group (BMSCs were plated in 6-well plates and 1.5 ml AKI-KHS was added to the upper chamber); the EPO-BMSCs + AKI-KHS group (EPO-BMSCs were plated in 6-well plates and 1.5 ml AKI-KHS was added to the upper chamber); the EPO-BMSCs + AKI-KHS + SIRT1 siRNA group (EPO-BMSCs transfected with SIRT1 siRNA were plated in 6-well plates and 1.5 ml AKI-KHS was added to the upper chamber); and the EPO-BMSCs + AKI-KHS + Con siRNA group (EPO-BMSCs transfected with control siRNA were plated in 6-well plates and 1.5 ml AKI-KHS was added to the upper chamber). All groups were incubated at 37 °C for 24 h in a humidified atmosphere of 5% CO_2_. Then cells were harvested for western blot analysis. After that, we established AKI-KHS-induced in vitro injury model again, the apoptosis of the BMSCs and EPO-BMSCs was analyzed by flow cytometry.

### Animals

Adult female SD rats (200–250 g), purchased from the animal house of the Faculty of Medicine at Southern Medical University (Guangzhou, China), were used for the study. Animals were housed under specific pathogen-free conditions with a 12 h dark–light cycle and supplied with pelleted food and tap water ad libitum. Animals were allowed to acclimatize to the housing conditions for 1 week. All procedures were performed in strict accordance with the Guidelines for Animal Experimentation of Southern Medical University (Guangzhou, China).

### Animal grouping and treatment

AKI models were established using SD rats. The animals were anesthetized by intraperitoneal injection of 2% sodium pentobarbital (50 mg/kg). Abdominal incisions were made, and the two renal pedicles were bluntly separated. A microvascular clamp was used to clamp both renal pedicles for 45 min, followed by clamp-release to allow reperfusion. Then, the abdominal incision was closed. Intervention treatments were administered to rats through tail vein injection after reperfusion.

To determine the effects of BMSCs and EPO-BMSCs treatment, we randomly divided the rats (n = 50) into the following five groups (10 rats per group): sham group (kidneys of the SD rats were exposed for 45 min and 1 ml low-glucose DMEM was injected), model group (both renal pedicles were clamped for 45 min and 1 ml low-glucose DMEM was injected), EPO group (both renal pedicles were clamped for 45 min and 1 ml EPO (500 IU/ml) was injected), BMSCs group (both renal pedicles were clamped for 45 min and 1 × 10^6^ BMSCs were injected), and EPO-BMSCs group (both renal pedicles were clamped for 45 min and 1 × 10^6^ EPO-BMSCs were injected). All rats were housed at a favorable temperature and humidity (a temperature of 21 °C ± 2 °C, and a humidity of 55% ± 5%) with an unlimited supply of water and food post-surgery. Rats were sacrificed 1 and 5 days after treatment (five rats at each time point). Blood samples and tissues from the kidneys, lung, spleen, and liver were collected for subsequent experiments. Blood samples were collected through the inferior vena cava, and serum was separated and stored at − 80 °C until use. Both kidneys of each rat were immediately excised and cut into two coronal sections. The sections were fixed in 4% paraformaldehyde at room temperature. In the BMSCs and EPO-BMSCs group, the lung, spleen, liver, and the remaining part of the kidney were immediately analyzed.

### GFP fluorescence in frozen tissue sections

In our previous study, we demonstrated that pretreatment with 500 IU/ml EPO for 48 h prior to infusion markedly increased the homing abilities of BMSCs. To verify the in vivo distribution of BMSCs after intravenous infusion, we prepared fast-frozen sections from the kidney, lung, spleen, and liver 24 h after GFP-BMSCs and GFP-EPO-BMSCs infusion. We immediately observed the sections under a fluorescence microscope.

### Renal functional analysis

Renal function was estimated using diagnostic kits. SCr was measured using a colorimetric microplate assay based on the Jaffe reaction (Quantichrom Creatinine Assay; BioAssay Systems). BUN was measured using a colorimetric assay kit according to the manufacturer’s instructions (Quantichrom Urea Assay; BioAssay Systems).

### ELISA

Serum concentrations of the inflammatory factors IL-1β and TNF-α and the anti-inflammatory cytokine IL-10 were measured by ELISA (R&D Systems, Minneapolis, MN, USA). The assay was performed according to the manufacturer’s instructions.

### Renal histological analysis

To detect kidney injuries, we fixed the samples in 4% neutral-buffered paraformaldehyde for histological assessment, embedded them in paraffin, and cut them in 3-μm-thick slices. The sections were then stained with HE (Servicebio, China). Histological examinations were performed in a blinded manner for acute tubular necrosis (ATN) scores regarding the grading of tubular necrosis, cast formation, tubular dilation, and loss of brush border. Ten non-overlapping fields (× 200) were randomly selected and scored as follows: 0, no damage; 1, patchy isolated necrosis ≤ 10%; 2, tubular necrosis between 10 and 25%; 3, tubular necrosis between 25 and 50%; 4, tubular necrosis > 50% [3].

### Statistical analysis

Results are expressed as mean ± standard deviation. Student’s *t* test was performed to analyze the differences between two groups. Multiple-group comparison was performed using one-way analysis of variance (ANOVA) test. SPSS 19.0 statistical software was used for statistical analysis. P < 0.05 was considered statistically significant.

## Data Availability

Not applicable.
